# Struggling to be Fit: Identity, Integrity, and the Law

**DOI:** 10.2966/scrip.140217.326

**Published:** 2017-12

**Authors:** Shawn HE Harmon, Abbe Brown, Sita Popat, Sarah Whatley, Rory O’Connor

**Affiliations:** * Honorary Fellow, School of Law, University of Edinburgh, Scotland; ** Professor in Intellectual Property Law, School of Law, University of Aberdeen, Scotland; *** Professor in Performance and Technology, School of Performance and Cultural Industries, University of Leeds; **** Professor of Dance, Centre for Dance Research, Coventry University; ***** Charterhouse Professor of Rehabilitation Medicine, Faculty of Medicine and Health, University of Leeds

**Keywords:** avatar, disability, identity, integrity, personhood, prosthetic

## Abstract

This interdisciplinary co-authored Analysis piece introduces identity and integrity, which are argued to sit at the core of the person. It analyses approaches taken to these concepts by legal regimes, particularly in the context of individuals using artificial limbs or digital avatars. The piece concludes that law engages with identity and integrity to a limited and incomplete extent; and that law is thus inadequate in its engagement with the person, and its meaning making in this respect. This piece draws on two interdisciplinary funded projects, funded by the Wellcome Trust and the Arts and Humanities Research Council.

## 1 Introduction

Law is one of the avenues by which we make meaning. It is, of course, a special avenue – or social institution – insofar as it contains strong purposive and coercive components. Law can facilitate or prohibit activities, and provide enforcement pathways for those facilitations and prohibitions. In so doing, law makes (and signals, though not always explicitly) value judgements about the utility and/or virtue of things, and of course, of ways of being. Law exposes, and helps remake, and sometimes embeds or ossifies social norms. In short, the law, through its expressiveness (i.e. through the oral and textual expressions that it makes via treaties, legislation, court decisions, and practices), is a core shaper of social expectations and behaviours.^1^ In addition to law, there are other avenues by which we give meaning to goods, to actions, to institutions, and to individuals, and by which we revise that meaning, and so by which we understand our reality.^2^

Against this backdrop, in this Analysis we offer a non-exhaustive survey of two concepts that are key to the development of the person, namely ‘identity’ and ‘integrity’. We explore how the law approaches or understands them, particularly regarding their impact on those with physical disabilities. We draw heavily on our research in the disability setting, specifically involving disabled dance artists in the Arts and Humanities Research Council funded “InVisible Difference Project”^3^ (which has roots deep in the history of *SCRIPTed*),^4^ and users of prosthetics limbs and gamers with digital avatars in the Wellcome Trust-funded “Identity and Governance of Bodily Extensions Project” (IGBE).^5^ These projects are both interdisciplinary, with empirical elements. InVisible Difference explored the relationship between dance, disability, and law and the need for legal, policy, and practical change; and IGBE explores the concept of extensions of self (both through artificial limbs and through digital avatars), with team members from law, performance, medicine, psychology, and sociology.

Our modest intent in this Analysis is to uncover the extent to which (some) laws are sensitive to the identity and integrity of persons, particularly those who use prosthetic limbs. First, we unpack the notions of identity and integrity as central to “meaning-making”, both generally and in the disability context.^6^ Equipped with the lens that these concepts provide, we then locate them in the broader legal landscape, offering an overview of the extent to which they are noticed, and how they are understood. This will also allow us to draw some preliminary conclusions about the extent to which the law empowers persons – and specifically prosthetic users – to act and participate in society.

## 2 Identity and Integrity: an introduction

In our previous interdisciplinary research,^7^ we explored the extent to which lived experiences of a person are intertwined with something which might be considered distinct from the person (e.g. a person controlling a digital avatar through a graphics tablet and experiences the avatar as being an extension of their own body); the links between a person’s dignity and their belonging to a wider community (such as that of disabled dance); and the need for a new approach to theorising dance made and performed by disabled dance artists. We concluded that the concepts of identity and physical integrity can be key to meaning-making in these contexts, and more generally so regarding respect for the person.

This also draws on other scholarship relating to identity and its importance to the person. Identity is a contested and multifaceted concept, and it has subjective and objective elements.^8^ Subjectively, “identity” can describe a variety of elements such as core personal values and self-perceptions, and objectively it can cover public statuses assigned at birth or later, and also how we might be described by others. Identities, then, can be internal and fluid, and also external and more permanent.^9^ These factors can apply to all persons; however, they are of particular relevance to prosthetic limb users. Empirical research carried out in the IGBE project and explored in more detail in another output (which also explored identity and integrity in more depth) found that the identities of prosthetic limb users are often deeply entangled with the external device or body extension that is the prosthetic.^10^

A different connection exists between the prosthetic limb user and integrity. Scholarship shows “integrity” to have a moral focus, drawing on completeness; it also has links with different forms of insult or causing offence.^11^ Integrity is relevant here as prosthetic limb users’ physical integrity is often seen as lacking or having been undermined by their condition (here the absence or one or more limbs), and there is a perceived lack of wholeness.^12^ Conversely, however, there is a rich body of work arguing that physical integrity need not involve a so-called “normal” body; if one is born, say, with one hand, then having a body with one-hand is normal and the body has integrity – the body is, however, non-normative.^13^

Strong arguments exist, then, for identity and integrity to exist as concepts, and for them to be particularly relevant (in potentially conflicting ways) to the development and personhood of prosthetic limb users. From this theoretical and empirical base, we focus in this piece on some instances of how law engages (or does not engage) with these concepts.

## 3 Identity and Integrity: and the Law

### 3.1 Identity

First, we acknowledge that the concept of “identity” is known to the law, but law’s interaction with the concept is often either indirect or non-explicit, or both. For example, the law acknowledges and shapes a range of phenomena that are pertinent to identity; it both confers and places restrictions on the rights available to certain groups based on identity-relevant factors such as developmental status (i.e. the right of foetuses to legal standing and protection);^14^ sexual orientation (i.e. the right to marry,^15^ the right to work benefits);^16^ and gender (i.e. the right to be recognised as the sex/gender with whom they most identify).^17^ Rather unexplored notions of identity have also been explicitly referenced by courts to inform ethical and legal arguments relating to kinship and new technologies such as in vitro fertilisation technologies. An example is *Rose and another v Secretary of State for Health*,^18^ in the context of an application for judicial review (regarding disclosure of information about artificial insemination under a legislative regime which did not provide for this). The court found that information about biological identity went to the heart of identity and to the make-up of the person; and that identity included details of origins and opportunity to understand them, physical and social identity, and also psychological integrity.

Identity is also explicitly referenced or unavoidably implicated in a range of legal instruments. Most notable is the European Convention on Human Rights (ECHR), which erects rights in respect of private life (which was discussed in *Rose*), religion, and expression.^19^ From a disability perspective, the International Convention on the Rights of Persons with Disabilities (2006) (CRPD) provides for respect for the right of children with disabilities to preserve their identities,^20^ and General Comments^21^ in respect of it explore layers of identity in the context of discrimination^22^ and linguistic identity in the context of the deaf.^23^

A review of legal instruments and usage, however, also demonstrates that identity is not understood uniformly. For example, the UN Convention on the Rights of the Child (1989) states that the child shall be registered immediately after birth and shall have the right from birth to a name, to acquire a nationality, and to know and be cared for by their parents.^24^ Further, the parties undertake to respect the right of the child to preserve his or her identity, including nationality, name, and family relations without unlawful interference and also that, where a child is illegally deprived of some or all of the elements of his or her identity, parties shall provide appropriate assistance and protection, with a view to re-establishing speedily his or her identity.^25^ Here, identity is associated with social connections (familial and community) insofar as they link to wellbeing, benefits, and traceability.

By contrast, the Convention on Human Rights and Biomedicine (1997) states that parties shall protect the dignity and identity of all human beings, and guarantee everyone, without discrimination, respect for their integrity and other rights and fundamental freedoms with regard to the application of biology and medicine.^26^ Here, identity is linked to dignity and the capacity to hold interests and rights that create space to make personal decisions, and so is closely associated with autonomy. Finally, the UNESCO International Declaration on Human Genetic Data (2003) explicitly recognises the special nature of genetic data to individual and group identity, and emphasises it as a critical factor in meaning-making. It goes on to provide, however, that a person’s identity should not be reduced to genetic characteristics (because it involves complex educational, environmental and personal factors and emotional, social, spiritual and cultural bonds with others and implies a dimension of freedom).^27^ So again, identity as characterised by genetic characteristics and kinship is recognised as important. In contrast, the work of the UN Rapporteur on the Rights of Persons with Disabilities (an appointment with, broadly, a mandate to lead and stimulate discussion and make recommendations regarding implementation)^28^ makes no reference to identity. Interestingly, however, the Rapporteur does focus on participation, inclusiveness, embracing diversity and change in social perceptions all points which can be argued to be relevant to identity.^29^

Law also provides opportunities for claims to be raised in respect of image, publicity, or personality rights, notably in respect of inaccurate claims of product endorsement and merchandising.^30^ The legal constructions for these rights are complex, drawing on the doctrine of passing off and Roman-Dutch principles of *iniuriam* – which builds in turn on Grotius’ claim that a man’s life is his own by nature, not indeed to destroy, but to preserve it, and so is his body, his limbs, his reputation, his honour, and his actions.^31^ This strand is another means of protecting identity. It requires, however, that necessary thresholds are met, notably that there is some form of reputation or goodwill (distinct from ownership but arguably analogous here), and then some activity and risk of damage.^32^

### 3.2 Integrity

Integrity is also encompassed by a range of human rights found in regional and international instruments. For example, the ECHR right to respect for private and family life discussed above has been held to “encompass moral and physical integrity and to extend to situations of deprivation of liberty”.^33^ There are also references to integrity in the CRPD and this is developed in General Comments.^34^ The CRPD provides that “[e]very person with disabilities has a right to respect for his or her physical and mental integrity”.^35^ Further, the CRPD provides that states are to take all steps to protect persons with disabilities from all forms of exploitation.^36^ Finally, the Universal Declaration on Bioethics and Human Rights (2006) provides that in advancing science and technology, there is to be respect for the personal integrity of people with special vulnerability.^37^

At the national level and moving away from physical disability, integrity also informs the rights of those who lack capacity (i.e. who cannot exercise autonomy) to be protected, and to have decisions taken on their behalf only in support of their best interests. For example, the Mental Capacity Act 2005, which applies to England and Wales, articulates the key theme of “best interests” (i.e. decision-makers must take decisions on someone’s behalf that are in that person’s best interest as understood from the perspective of that person). The legislation also has a theme of “least restrictive means” (i.e. where a decision is taken that interferes with the person’s physical integrity, the option that represents the least restrictive means must be adopted – it must impose on them in a limited way and the idea of proportionality is important).^38^ When applying these tests (outside the prosthetic context), courts have seen physical integrity as important.^39^

## 4 A different perspective: disability

### 4.1 Law and disability

We can see, then, that the law does engage both directly and indirectly with the concepts of identity and integrity, which are so important to meaning-making, and so to personal narrative and worldview. However, we have seen that the law’s reliance on them is not equal, nor is the law’s understanding of them consistent. Further, the position of these concepts in specific legal regimes discussed (including those relating to disability) does not reflect the importance accorded to these concepts by prosthetic users as indicated by our empirical research and the scholarly arguments discussed above. In fact, when it comes to prosthetic users (and disabled persons more generally), the law engages more readily with the concept of disability. In so doing, however, it will be suggested that an overly narrow and problematic approach has been taken, which does not engage with the importance of the person.

International human rights instruments provides that everyone is entitled to dignity,^40^ and to certain economic, social and cultural rights which are viewed as indispensable to the achievement of dignity and the free development of personality.^41^ However, the focus of service-provision and rights-protection under these instruments has, in the main, been on ensuring survival, access, and participation.^42^ We have not seen a celebration of diversity, facilitating achievement of excellence (which has been achieved by people with disabilities in areas such as in sport,^43^ dance,^44^ or comedy^45^), or seeking to enable true flourishing.^46^ Possible goals in this respect might be the requirement for elite funding across these areas^47^ and the inclusion of this in international reports. This wider approach would be more consistent with a focus on the person and on engaging with identity and integrity.

This would also have an impact at the domestic level. So far, the UK Equality and Human Rights Commission^48^ and the Scottish Human Rights Commission^49^ tend to focus on food, housing, and basic employment. Their 2017 reports to the Committee of the Convention on the Rights of Persons with Disabilities emphasise (appropriately) dignity and autonomy, but then focus on identifying barriers to good standards of independent living, safety and accessibility, health, and life.^50^ The reports do not take the opportunity to construct body diversity as “normal” and as having a central place in the mainstream.^51^ They do not engage robustly with true equality and social justice for the individual person and the body/identity link – in essence, looking beyond functionality to an approach more integrated with creative and cultural expression and achievement and wider growth, reflecting developments seen in the avatar context.^52^ The national position is also disappointing as the CRPD in its engagement with culture and sport refers to access, and to people with disabilities being able to fulfil their creative, artistic, and intellectual potential for their own benefit and also that of society.^53^ Accordingly, from this base, there remains the need for a strong call for true equality.^54^

### 4.2 Reflections

The narrow emphasis taken in the main to delivery of human rights at the international and national level may, arguably, be a natural consequence of the origins of human rights law (as a response to misdeeds perpetrated in World War II). It may also reflect widespread views on the appropriate limits of human rights (as tools for ensuring “freedom from” various activities by the state rather than anything more positive requiring substantial action or investment on the part of the state or others).^55^

The approach is also, arguably, a consequence of the “medical” model of disability that the law and powerful social actors (like the medical profession) have long held to and pursued. This sees disability as a problem to be cured.^56^ More recently, the “social” model of disability can be seen in the law. This model adopts the position that disability arises from social structures and environmental barriers.^57^ The Equality Act 2010, which is the UK’s response to the CRPD,^58^ contains hints of the social model with a focus on protected characteristics, which include disability.^59^ The legislation focuses on activities which a person may be able to do, or may be being prevented from doing because of their condition (again from the upright bipedal perspective); and the legislation imposes on the providers of services and also the public sector more generally (but not more widely) the obligation to make reasonable adjustments to practices and physical spaces to accommodate individuals with these characteristics – considering in detail employment, education, and property.^60^

The social model also underlies the Scottish Government’s 2016 “A Fairer Strategy for Disabled People”, which was built on strong engagement with disabled people.^61^ Yet the social model, in turn, has been challenged by the “affirmative” model. This model focuses on what people can do rather than on what they cannot do.^62^ And it is this mode which would enable greater (indirect) delivery of identity and integrity and regard to the person, through requiring, say, equality of funding and equality of programming of events and through which wider society could be transformed through inclusion.

## 5 Delivery

Finally, quite apart from substantive challenges to law’s engagement with integrity and identity, it should be noted that the ability to bring an action to pursue identity and integrity can be limited. One needs a claim that fits into a specific framework, such as a claim for privacy with the necessary requirements being met,^63^ a claim for paternity recognition,^64^ a demand for a particular activity to be prevented,^65^ or an application for judicial review as seen. The existence of national human rights legislation – such as the UK’s Human Rights Act 1998 – does not mean that the human rights discussed above are direct or easily accessed pathways to national courts.^66^ Further, the Equality Act 2010 does not impose obligations on all.

To address this, there should be an imposition of equality obligations on all at the national level; a right to pursue these obligations directly at court; and a clearer international requirement for countries to invest in and report on how they are supporting an affirmative approach to diversity (rather than merely enabling physical access and basic mobility). The first two would be a significant change given the place of human rights and treaties in the UK, and the third is inconsistent with the present international framework. All three are, however, consistent with the 2017 CRPD draft General Comment regarding equality and non-discrimination.^67^ And the proposals are necessary to enable law to provide an appropriate place for integrity, identity, and the person.

## 6 Conclusion

Identity and integrity have been identified by empirical and conceptual work as being at the core of the person. This analysis reveals, however, that they are not accorded such importance by the law. There are various legal tools by which one might seek to pursue or defend identity or integrity. The focus of the legal action, however, will not come under those terms; the relevant laws and pathways operate without a focus on the person. Law is failing, then, in its meaning making to address the person – indeed, the person is peripheral. This was a disappointing message for the lawyers to report to interdisciplinary colleagues; and in our ongoing work building on the InVisible Difference and IGBE projects, we argue for change in this respect. Three rather radical proposals have been made here as to how law can be adapted. This would create wider obligations to address inequality, more focus on existing human rights which address the whole of the person, and also on the person’s opportunities to engage across society. This would go some way to creating a new reality for, and meaning in respect of, the person. Law might not be willing to accommodate such radical proposals that run to the heart of disability and human rights legislation. If so, law will continue to fail in its meaning-making.

## Footnotes

1 Shawn Harmon, “Katri Lohmus*, Caring Autonomy: European Human Rights Law and the Challenge of Individualism”* (2017) 25 *Medical Law Review* 1-9. For a study on the expressive function of legislation, see Patricia Funk, “Is There an Expressive Function of Law? An Empirical Analysis of Voting Laws with Symbolic Fines” (2007) 9 *American Law and Economics Review* 135-159. For observations on the importance of the expressive functions of courts, see Bora Laskin, “The Role and Functions of Final Appellate Courts: The Supreme Court of Canada” (1975) 53 *Canadian Bar Review* 469; William Rehnquist, “The Changing Role of the Supreme Court” (1986) 14 *Florida State University Law Review* 1; Aharon Barak, “A Judge on Judging: The Role of a Supreme Court in a Democracy” (2002-2003) 119 *Harvard Law Review* 19; Joanne Scott and Susan Sturm, “Courts as Catalysts: Re-Thinking the Judicial Role in New Governance” (2006) 13 *Columbia Journal of European Law* 565-594; Neil Siegel “The Virtue of Judicial Statesmanship’ (2007) 86 *Texas Law Rev* 958-1032.

2 Steven Krauss, “Research Paradigms and Meaning Making: A Primer” (2005) 10(4) *The Qualitative Report* 758-770.

3 See Project website http://www.invisibledifference.org.uk/ (accessed 12 November 2017), which sought to extend thinking around the making, status, ownership and value of work by contemporary dance choreographers, focusing specifically on that made and performed by disabled dance artists.

4 See Abbe Brown, Shawn Harmon and Charlotte Waelde, “SCRIPT: A Legacy of Vitality” (2015) 12(1) *SCRIPTed* 51-58, available at https://script-ed.org/?p=1935 (accessed 12 November 2017).

5 See Project website http://www.pci.leeds.ac.uk/research/featured-research-projects/identity-and-governance-of-bodily-extensions-the-case-of-prosthetics-and-avatars/ (accessed 12 November 2017); Sita Popat, Sarah Whatley et al., “Bodily Extensions and Performance” (2017) 13(2) *International Journal of Performance Arts and Digital Media* 101-104; and Shawn Harmon et al., “Bodily Extension and the Law: Medical Devices, Intellectual Property, Prosthetics and Marginalisation (Again)” (manuscript in preparation).

6 There may well be other concepts of significance to prosthetic-users, but our research highlights these as particularly important, and they potentially have some resonance with the frameworks we are exploring.

7 Charlotte Waelde et al., *“*A New Foundation: Physical Integrity, Disabled Dance and Cultural Heritage” in Sarah Whatley et al. (eds.), *InVisible Difference: Dance, Disability and Law* (Bristol: Intellect, 2017 forthcoming); Sita Popat and Scott Palmer, “Embodied Interfaces: Dancing with Digital Sprites” (2008) 19(2) *Digital Creativity* 125-137.

8 Augusto Blasi and Kimberly Glodis, “The Development of Identity: A Critical Analysis from the Perspective of the Self as Subject” (1995) 15 *Developmental Review* 404-433.

9 Scott Lasch and Jonathan Friedman (eds.), *Modernity and Identity* (Basel: Blackwell, 1992); Donald Polkinghorne, “Explorations of Narrative Identity” (1996) 7 Psychological Inquiry 363-367; Leonie Huddy, “From Social to Political Identity: A Critical Examination of Social Identity Theory” (2001) 22 *Political Psychology* 127-156. For the construction of identity in specific settings, see e.g. Ingegard Fagerberg and Mona Kihlgren, “Experiencing a Nurse Identity: The Meaning of Identity to Swedish Registered Nurses 2 Years After Graduation” (2001) 34 *Journal of Advanced Nursing* 137-145; Elisa Abes and David Kasch, “Using Queer Theory to Explore Lesbian College Students' Multiple Dimensions of Identity” (2007) 48 *Journal of College Student Development* 619-636; Jesse Smith, “Becoming an Atheist in America: Constructing Identity and Meaning from the Rejection of Theism” (2011) 72 *Sociology of Religion* 215-237.

10 Harmon et al. (manuscript in preparation), *supra* n. 5.

11 Axel Honneth, “Integrity and Disrespect: Principles of a Conception of Morality on the Theory of Recognition” (1992) 20 *Political Theory* 187-201; Roberta Galler, “The Myth of the Perfect Body” in Carole Vance (ed.) *Pleasure and Danger: Exploring Female Sexuality* (London: Routledge & Kegan Paul, 1984) 165-172; Charlotte Waelde et al., “A New Foundation”, *supra* n. 7.

12 Tomoko Tamari, “Body Image and Prosthetic Aesthetics. Disability, Technology and Paralympic Culture” (2017) 23(2) *Body & Society* 25-56; Shawn Harmon, Kate Marsh, Sarah Whatley and Karen Wood, “Moving toward a new aesthetic” in Sarah Whatley et al. (eds.), *InVisible Difference*, *supra* n. 7.

13 Galler, *supra* n. 11; Sharon Betcher, “Putting my Foot (Prosthesis, Crutches, Phantom) Down: Considering Technology as Transcendence in the Writings of Donna Harraway” (2001) 29 *Women’s Studies Quarterly* 35-53.

14 *Vo v France* (2005) 40 EHRR 12. For more examples, see Aurora Plomer, “A Foetal Right to Life? The Case of Vo v France” (2005) 5 *Human Rights Law Review* 311-338.

15 International Covenant on Civil and Political Rights (1966) (hereafter ‘ICCPR’), art 23(2); Ignacio Saiz, “Bracketing Sexuality: Human Rights and Sexual Orientation: A Decade of Development and Denial at the UN” (2004) 7 *Health and Human Rights* 48-80.

16 International Covenant on Economic, Social and Cultural Rights (1966) hereafter ‘ICESCR’, arts 6(1), 7.

17 On basis European Convention on Human Rights and Fundamental Rights (1951) (hereafter ‘ECHR’), art 8 right to private life, see also *Goodwin v United Kingdom* (28957/95) [2002] I.R.L.R. 664.

18 *Rose and another v Secretary of State for Health* [2002] EWHC 1593 (Admin).

19 Although note that each have their own limits. For more on rights and identity, see Jill Marshall, *Personal Freedom through Human Rights Law? Autonomy, Identity and Integrity under the European Convention on Human Rights* (Leiden: Brill, 2008); Jill Marshall, *Human Rights Law and Personal Identity* (London: Routledge, 2014); Romina Sijniensky and N Alexander Aizenstatd, “Towards a Uniform Basis for the Right to Identity in the Normative Framework of the American Convention on Human Rights’ (2014) 7 *Inter-American and European Human rights Journal* 75-92, in the context of disappearing children.

20 Convention on the Rights of Persons with Disabilities (2006) (hereafter ‘CRPD’), art 3(h).

21 See Committee on the Rights of Persons with Disabilities General Comments webpage, http://www.ohchr.org/EN/HRBodies/CRPD/Pages/GC.aspx (accessed 12 November 2017).

22 CRPD General Comment No. 3 considering intersectional discrimination, building on layers of identities, and Daniela Ikawa, “The Construction of Identity and Rights: Race and Gender in Brazil” (2014) 10 *International Journal of Law in Context* 494-506.

23 CRPD General Comment No. 4 regarding inclusive education, arts 9, 34 (b), 56. See also draft CRPD General Comment No. 6 (April 2017) on CRPD, art 5 right of persons with disabilities to equality and non-discrimination, available at http://www.ohchr.org/EN/HRBodies/CRPD/Pages/CallPersonsDisabilitiesEqualityResponsability.aspx (accessed 12 November 2017) paras 10 and 11.

24 UN Convention on the Rights of the Child (1989) (hereafter ‘CRC’) art 7(1).

25 CRC, art 8.

26 Convention for the protection of Human Rights and Dignity of the Human Being with regard to the Application of Biology and Medicine: Convention on Human Rights and Biomedicine (1997), art 1.

27 UNESCO International Declaration on Biological Human Data (2003), recital 6, art 3, 4.

28 Appointed 2014, see UN Human Rights Officer of the High Commissioner Webpage http://www.ohchr.org/EN/Issues/Disability/SRDisabilities/Pages/SRDisabilitiesIndex.aspx (accessed 12 November 2017).

29 UN General Assembly. Human Rights Council. 28^th^ session. A/HRC/28/58. Available at http://www.ohchr.org/EN/HRBodies/HRC/RegularSessions/Session28/Pages/ListReports.aspx (accessed 12 November 2017), paras 4, 19(a) (b) (c), 33(c).

30 *Mirage Studios v Counter-Feat Clothing Co Ltd* [1991] FSR 145; *Irvine v Talksport Ltd* [2002] EWHC 367 (Ch.); *Fenty v Arcadia Group Brands Ltd (t/a Topshop)* [2015] EWCA Civ 3; *OBG Ltd v Allan* [2007] UKHL 21.

31 H Grotius (transl. A Campbell), *De juri belli ac pacis* Lib. II, Cap. XVII, II (NY: M Walter Dunne, 1901).

32 See generally Gillian Black, *Publicity Rights and Image: Exploitation and Legal control* (Oxford: Hart, 2011); Elspeth Christie Reid, *Personality, Confidentiality and Privacy in Scots Law* (W Green, 2010), 5-14; *Grutter v Lombard* (628/05) [2007] ZASCA 2. South Africa has used the question of identity in passing off and image personality type cases (*ibid*., paras 7 – 13); this is the converse of a developing debate in the law of passing off regarding how can brand owners protect their goodwill (for well-known celebrities arguably their identity) when it is being used beyond particular sets of goods. Here, the focus of actual self being extended, rather than its power being extended. See Catherine Ng, “The Law of Passing Off – Goodwill Beyond Goods” (2016) *International Review of Intellectual Property and Competition Law* 817-842.

33 *Husayn v Poland* (2015) 60 EHRR 16 (ECtHR). See also *Dickson v UK* (2008) 46 EHRR 41 (Grand Chamber).

34 References are made to integrity in draft CRPD General Comment No. 6 paras 5, 8, supra n 23.

35 CRPD, art 17 also considered in CRPD General Comment No. 1 (regarding CRPD art 12 right to equal recognition before the law) paras 29, 31, 42.

36 CRPD, art 16(1).

37 Universal Declaration on Bioethics and Human Rights (2006), art 8.

38 Mental Capacity Act 2005, s 1.

39 See *A Local Authority v K* [2013] EWCOP 242 (Fam) regarding a person with learning disabilities and sterilisation as opposed to a coil para 26; *Aintree University Hospital NHS Foundation Trust v James* [2013] 3 WLR 1299 (SC) paras 19 et seq regarding withdrawal of treatment; and *Mental Health Trust and others v DD* [2015] EWCOP 4 (Fam) also on contraception and sterilisation paras 5, 84.

40 Universal Declaration of Human Rights (hereafter ‘UDHR’) (1948), art 1.

41 See UDHR, art 22. There are also treaty references to culture, freedom of expression, and creative achievement: ICESCSR, art 15(1)(a) and (c); ICCPR, art 19; CRPD, arts 21 and 30.

42 See UN Committee on Economic Social and Cultural Rights General Comment No. 5 (1994) paras 37-9 and UN Committee on Economic, Social and Cultural Rights General Comment No. 21 (2009) para 30.

43 E.g. Paralympics at Rio 2016, available at https://www.paralympic.org/rio-2016 (accessed 12 November 2017), notably the achievements of Jonnie Peacock and Emma Wiggs.

44 E.g. Caroline Bowditch, available at http://www.carolinebowditch.com and Claire Cunningham http://www.clairecunningham.co.uk, (both accessed 12 November 2017).

45 E.g. The Last Leg, available at http://www.channel4.com/programmes/the-last-leg (accessed 12 November 2017).

46 Marshall, *Personal Freedom*, supra n 19**,** pp 25, 26 considering Conor Gearty *Can Human Rights Survive?* (Cambridge: CUP, 2006) in particular pp 49, 50, 58, 141.

47 E.g. building on UN Committee on Economic Social and Cultural Rights General Comment No. 21 (2009) para 52 (d) regarding financial assistance, although this does not refer to disability.

48 Equality and Human Rights Commission, available at https://www.equalityhumanrights.com/en (accessed 12 November 2017).

49 Scottish Human Rights Commission, available at http://www.scottishhumanrights.com/ (accessed 12 November 2017).

50 UKEHRC, *Disability Rights in the UK: UK Independent Mechanism Submission to Inform the CRPD - List of Issues on the UK* (2017) http://www.scottishhumanrights.com/media/1559/crpdfeb2017ukimuksubmission.pdf (last accessed 12 September 2017); UKEHRC, *Disability Rights in Scotland: Supplementary Submission to Inform the CRPD - List of Issues on the UK* (2017), at http://www.scottishhumanrights.com/media/1558/crpdfeb2017scotlandsupplement.pdf (last accessed 12 September 2017).

51 For a wider discussion of disability and law, see Aileen McColgan, *Discrimination, Equality and the Law* (Oxford: Hart, 2014).

52 Given this narrow focus, it also interesting to note that debates have recently commenced around the creation of new rights to encompass the digital world and to better reflect our position(s) within it. See claims to rights of access to the internet (Labour digital manifesto, available at http://www.jeremyforlabour.com/digital_democracy_manifesto, and Nicola Lucchi, *The Impact of Science and Technology on the Rights of the Individual* (New York: Springer, 2016), pp. 31-96) and, in the light of the discussions above regarding digital avatars, note Jack Balkin and Beth Noveck (eds.), *The State of Play: Law, Games and Virtual Worlds* (New York: NYU Press, 2006), and Ralph Koster “A Declaration of the Rights of Avatars”, available at http://www.raphkoster.com/games/essays/declaring-the-rights-of-players/) (accessed 12 November 2017).

53 CRPD, arts 30(1), (2), (5).

54 For wider consideration of this, engagement with dance and the possible place of NGOs in CRPD monitoring, see Catherine Easton, “An Analysis of Reporting and Monitoring in Relation to the United Nations Convention on the Rights of Persons with Disabilities, the Right to Participation in Cultural Life and intellectual property” in Sarah Whatley et al. (eds.), *InVisible Difference*, *supra* n. 7.

55 As such, economic and cultural rights were long seen as aspirational rather than enforceable. See Katherine Young, “The Minimum Core of Economic and Social Rights: A Concept in Search of Content” (2008) 33 *Yale Journal of International Law* 113-17; Tamara Hervey and Jeff Kenner (eds.), *Economic and Social Rights Under the EU Charter of Fundamental Rights: A Legal Perspective* (Oxford: Hart, 2003). However, such a narrow approach to human rights is increasingly questioned. See Jerome Bickenbach et al., “Models of Disablement, Universalism and the International Classification of Impairments, Disabilities and Handicaps” (1999) 48 *Social Science & Medicine* 1173-1187; Rachel Hurst, “The International Disability Rights Movement and the ICF” (2003) 25 *Disability and Rehabilitation* 572-576.

56 See discussion in UPIAS *Fundamental Principles of Disability* (London: Union of the Physically Impaired Against Segregation, 1976) and Bradley Areheart, “When Disability isn't just Right: The Entrenchment of the Medical Model of Disability and the Goldilocks Dilemma” (2008) 83 *Indiana Law Journal* 181-232.

57 Colin Barnes and Mike Oliver, “Disability Rights: Rhetoric and Reality in the UK” (1995) 10(1) *Disability and Society* 111-116; Colin Barnes “The Social Model of Disability: Valuable or Irrelevant” in Nick Watson et al. (eds.), *The Routledge Handbook of Disability Studies* (London: Routledge, 2012) pp. 12-29.

58 Consider CRPD, preamble c; compare ICCPR, art 2(1) and ICESCR art 2(2). Note also Colin Barnes and Geoffrey Mercer (eds.), *The Social Model of Disability: Europe and the Majority World* (Leeds: The Disability Press, 2005).

59 See Equality Act 2010 (hereafter ‘EA 2010’), chapter 1 and s 6.

60 See EA 2010, s 1, 20, 29, 36 et seq, 39 et seq, 84 et seq 149, Schedules 2-4. See also reflections in the work of the Rapporteur, see Human Rights Council supra n 29.

61 Scottish Government, “Our Delivery Plan to 2021 for the United Nations Convention on the Rights of Persons with Disabilities”, available at http://www.gov.scot/Publications/2016/12/3778/5 (accessed 12 November 2017).

62 John Swain and Sally French, “Towards an Affirmation Model” (2000) 15(4) *Disability and Society* 569-582. See also discussion in Abbe Brown and Charlotte Waelde, “Human rights, Persons with Disabilities and Copyright” in Christophe Geiger, (ed.), *Research Handbook on Human Rights and Intellectual Property* (Cheltenham: Edward Elgar, 2015) pp. 577-602.

63 *Campbell v MGN* [2004] 2 All ER 995 (HL); *Weller v Associated Newspapers* [2016] 3 All ER 357 (CA).

64 *Mandet v France*, Application No. 30955/12, 14 January 2016, ECtHR (Fifth Section), reported in French.

65 Such as criminal digital profiling. See *Marper v United Kingdom* (30562/04), (2009) 48 EHRR 50; *Z v Finland* (22009/93) (1998) 25 EHRR 371; Karolina La Fros-Owczynik, “Minor Protection nor Major Injustice? – Children’s Rights and Digital Preventions Directed at Youth in the Dutch Justice System” (2015) 31(5) *Computer Law & Security Review* 651-667. Regarding digital identity more generally, see Clare Sullivan and Sophie Stalla-Bourdillon, “Digital Identity and French Personal Rights – A Way Forward in Recognizing and Protecting an Individual’s Rights in his/her Digital Identity”(2015) 31(2) *Computer Law & Security Review* 268-279; Roger Brownsword, “Friends, Romans, Countrymen: Is There a Universal Right to Identity?” (2009) 1(2) *Law, Innovation and Technology* 223-249.

66 See Human Rights Act 1998, ss3 and 6; ECHR, art 34; *Spencer v United Kingdom* (1998) 25 EHRR CD105; and Optional Protocol to CRPD (2006), in force May 2008.

67 See draft CRPD General Comment No. 6 supra n 23.

